# Imaging of Atrioventricular Nodal Conduction Tissue in Porcine Hearts Using Optical Coherence Tomography

**DOI:** 10.19102/icrm.2019.100601

**Published:** 2019-06-15

**Authors:** Orhan U. Kilinc, Xiaowei Zhao, Michael W. Jenkins, Christopher S. Snyder, Andrew M. Rollins

**Affiliations:** ^1^Congenital Heart Collaborative, Rainbow Babies and Children’s Hospital, University Hospitals, Cleveland, OH, USA; ^2^Department of Biomedical Engineering, Case Western Reserve University, Cleveland, OH, USA

**Keywords:** Atrioventricular node, cardiac electrophysiology, catheter ablation, imaging

## Abstract

Optical coherence tomography (OCT) employs near-infrared light to image the microstructure of different tissues. Clinically, it has been used to image the walls of coronary arteries. In research settings, one of the applications for OCT is visualizing endocardial and subendocardial structures. The present experiment sought to determine whether OCT can identify native conduction tissues in adult porcine hearts. During the study, the right atrial endocardial surfaces of excised adult porcine hearts were exposed. The triangle of Koch was imaged with the OCT system and the conduction tissue was identified. The area was then prepared for histologic examination with Masson’s trichrome stain. The results of histologic preparations and OCT images were then compared. Ultimately, nine porcine hearts were examined using this methodology. OCT imaging successfully identified subendocardial structures presumed to be the compact atrioventricular node. Histologic images of the preparations delineated the different tissue types and conduction tissue was easily identified. The location of distinctive hyporeflective areas in the OCT images correlated with the location of conduction tissue in the histology images. In light of the findings of this study, it is suggested that atrioventricular nodal tissue can be identified by OCT in freshly dissected unfixed porcine hearts. OCT images distinguished the differentiated conduction tissue in close proximity with the endocardium, myofibers, and fibrous tissue, and the success of this was verified with histology. This technology may be useful for the direct visualization of the native conduction system during procedures in the operating room and electrophysiology laboratory. Further studies with perfused tissue samples and live animal experiments are needed to better assess the efficacy of this novel application.

## Introduction

Radiofrequency ablation (RFA) and cryoablation (CA) are the preferred invasive treatment modalities for the management of pediatric arrhythmias.^1^ RFA was first described in 1981 and has since been refined to be a highly effective procedure with low associated complication rates.^2–6^ However, there still remain some serious complications that can occur, including permanent damage to the native conduction tissue, resulting in atrioventricular (AV) block. This complication results in AV dissociation and most often necessitates the placement of a pacemaker. There is about a 1% risk of complete AV block in RFA procedures when the arrhythmia substrate is on or near the native conduction system.^7–9^ Separately, CA is a newer invasive treatment for arrhythmias that is now frequently utilized for arrhythmia substrates on or near the normal conduction tissue. The main advantages of CA are its slow lesion progression and the ability to create reversible lesions. To our knowledge, there have not been any documented cases of permanent AV block with CA. The major drawback of CA, however, is that patients treated with this approach boast higher recurrence rates in comparison with those treaded using RFA.^10–12^

Optical coherence tomography (OCT) is a near-infrared, light-based imaging modality. It has micrometer-level resolution with 1-mm to 3-mm penetration.^13^ OCT was first used to image the retina; subsequently, it gained popularity in the cardiology field for imaging coronary arteries after the development of catheter-based systems.^14^ OCT catheter probes use fiber optics to transfer light from the source and then detect the backscatter from the tissue. Current commercially available OCT systems use side-viewing probes, and rotation of the probe with simultaneous pull-back allows for the three-dimensional imaging of the walls of the coronary arteries.^15^ Forward-imaging probes, which utilize rotational scanning, have also been introduced,^16–18^ but so far these have not been implemented broadly in clinical use.

Previous studies have revealed OCT’s ability to image the Purkinje network^19^ and show myocardial fiber orientation in processed heart tissues.^20–22^ Visualizing the AV conduction tissue (ie, the AV node) prior to placing ablation lesions may reduce the risk of AV block during radiofrequency ablation. The purpose of the present study was to determine whether OCT can successfully identify the AV nodal tissue in porcine hearts.

## Methods

The experiments were conducted on domestic porcine hearts (taken from domestic pigs weighing 60–80 lbs). After a pig was anesthetized and euthanized, the heart was explanted by the surgical skills laboratory staff at our institution. The heart was then placed into a cold (+4°C) phosphate-buffered saline solution during transport between the laboratories. All procedures were reviewed and approved by the Institutional Animal Care and Use Committee.

**Figure 1:**
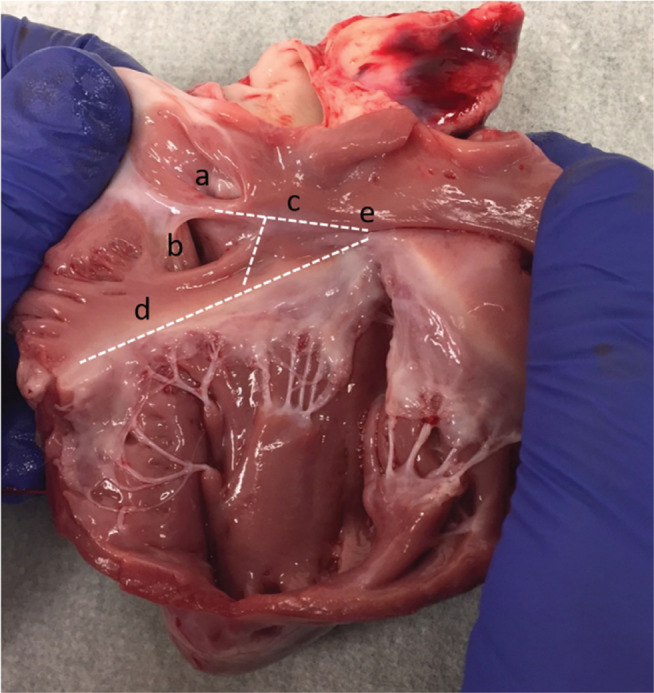
The triangle of Koch is the area bordered by the dotted lines, with the (a) fossa ovalis, (b) coronary sinus, (c) tendon of Todaro, (d) tricuspid valve annulus, and (e) central fibrous body pointed out.

To prepare the explanted heart, initially, a posterior incision was made in the right atrial wall and the anatomic structures were identified. Next, an anterior incision was made from the superior vena cava extending to the right ventricular apex, exposing the entire right atrial and ventricular endocardium **(Figure 1)**. After identifying the landmarks of the triangle of Koch, the specimen was carefully debulked for ease of manipulation and imaging.

**Figure 2:**
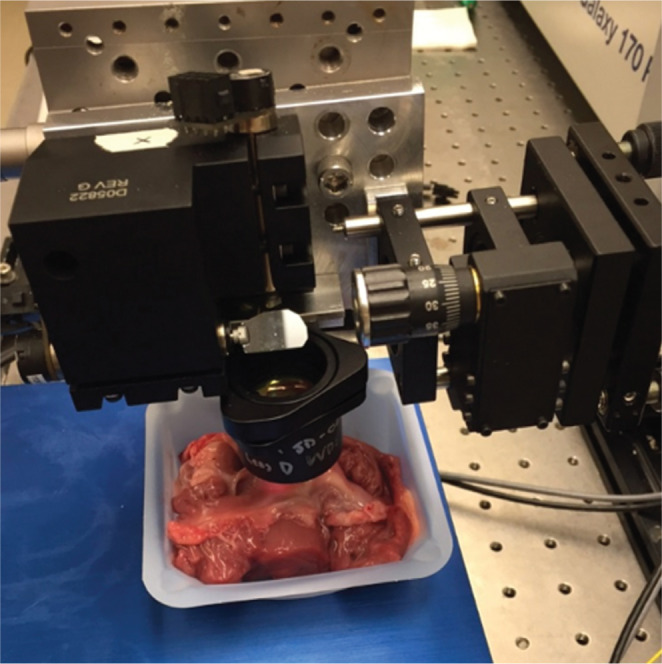
Benchtop spectral-domain OCT scanner.

The tissue samples were imaged using a noncontact, benchtop spectral-domain OCT system **(Figure 2)**. The axial and lateral resolutions of the scanner were both 12 μm and the imaging rate was 47,000 A-lines per second. For each sample, 800 × 800 lateral A-lines were recorded over an 8-mm × 8-mm region of interest, resulting in a three-dimensional image volume that could be reviewed in a slice-by-slice manner.

Based on the OCT system’s resolution, the OCT images were expected to correlate with histologic characteristics of the AV node.^23^ The subendocardium within the triangle of Koch and surrounding areas were examined with OCT during live scanning; once the nodal tissue was identified within the triangle of Koch, single-frame images were acquired. The identification was based on the shape of the tissue contrast between the nodal tissue, myocardium, and surrounding fibrous tissue (fibrous tissue is hyper-reflective and myocardium is less-reflective in comparison) and the location (ie, borders of the triangle of Koch). Since the AV node is surrounded by fibrous tissue, a hyporeflective area in the subendocardium, encapsulated by the hyper-reflective tissue, was considered as the identifier for the AV node.

For validation purposes, OCT images were compared with histology outcomes of the respective tissues. After identification of the AV nodal tissue with OCT, the field of view was marked with pins (100 μm in diameter) in the plane of the acquired OCT image. The tissue was then fixed in 10% formalin solution for 24 hours at 4°C for standard histological analysis (paraffin embedding with 5-μm sections). Masson’s trichrome stain was used to evaluate differentiation between the myocardium and connective tissue. Histologic stains were performed using the conventional technique in the core pathology laboratory of our institution. Slides were then examined via standard light microscopy performed using a Leica MF16F microscope (Leica Microsystems, Wetzlar, Germany) coupled with a Qimaging Retiga EXi camera (Quantitative Imaging Corp., Surrey, BC, Canada). Images were collected using the QCapture Pro 6.0 software (Quantitative Imaging Corp., Surrey, BC, Canada) and compared with the previously acquired OCT images. If the histology slide had the conduction tissue in it, fitting the description of the AV node, it was considered as correlating with the OCT image.

## Results

**Figure 3:**
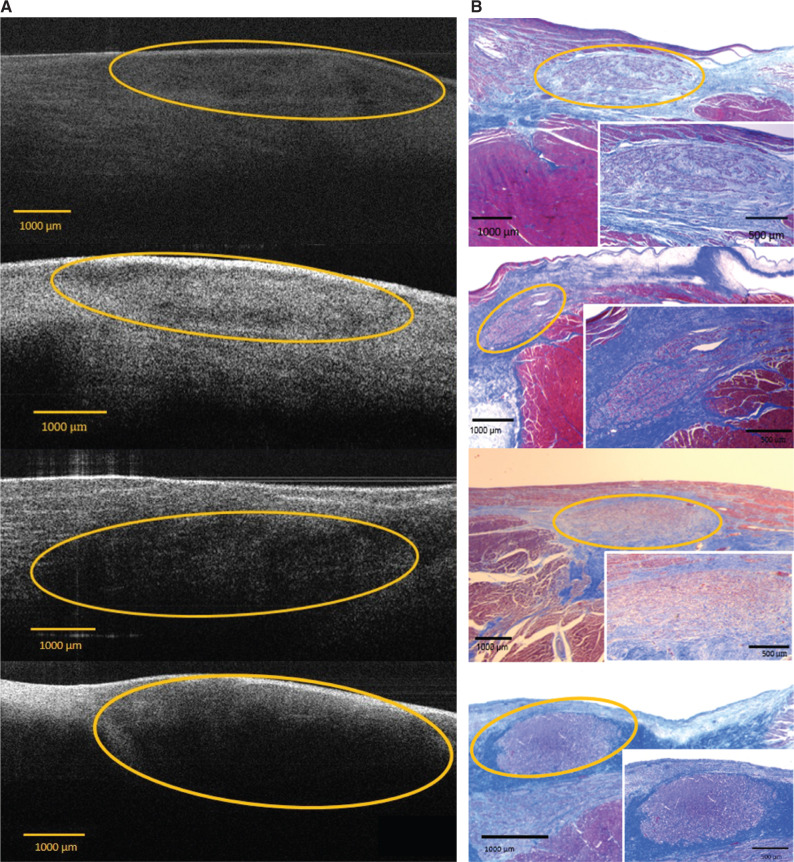
**A:** OCT images with the respective AV nodes marked with ovals. **B:** Corresponding AV node histology slides (low and high magnification), stained with Masson’s trichrome.

A total of nine pig hearts were examined. Cross-sectional OCT images in the triangle of Koch showed a spindle-shaped, hyporeflective, dark area in the subendocardium. This hyporeflective area was surrounded by a relatively bright, highly reflective area representing the fibrous tissue **(Figure 3A)**.

Staining the examined area with Masson’s trichrome revealed a portion of elliptical-/spindle-shaped tissue in close association with the connective tissue and the myofibers **(Figure 3B)**. Of note, this elliptical-/spindle-shaped tissue was almost entirely encapsulated by the connective tissue, and the staining pattern was distinct from the contractile myocardium. Higher magnification of this area showed cells with large nuclei and pale cytoplasm, surrounded by connective tissue, which are the characteristics of differentiated cells of AV nodal tissue.^23^

In all examined hearts, AV nodal tissue was identified using OCT imaging. The AV nodal tissue on OCT images correlated with the histology results, confirming that the OCT device was able to distinguish the AV node from the surrounding fibrous tissue and the myocardium.

## Discussion

OCT is gaining popularity in different fields of medicine by providing high-resolution, detailed images of small and delicate structures^24^; among other applications, endovascular use of OCT has been shown to be particularly useful.^14^ In 2002, Gupta et al. were able to show segments of AV nodal tissue after fixation.^20^ Our study proves that AV nodal tissue can be imaged by OCT in fresh tissue; this would likely mimic images obtained in vivo. Based on the differences in reflectivity, the contrast among the myocardium, fibrous, and nodal tissues in the triangle of Koch were clear in our experiments. This raises the possibility that OCT may be useful in identifying nodal tissue in vivo and thus is potentially applicative in clinical practice.

Importantly, intracardiac echocardiogram (ICE) and intravascular ultrasound (IVUS) are current other options for visualizing cardiac structures. ICE utilizes transducers with frequencies of between 5 MHz and 10 MHz and tissue penetration depths ranging from 1.5 cm to 5 cm.^25^ Because of the imaging properties, this modality is primarily used in interventional cardiology as an adjunct imaging method, specifically to identify macrostructures during catheter ablations.^26^ Although this imaging modality has deeper tissue penetration, the image resolution is not sufficient enough to visualize the subendocardium adequately. An integrated diagnostic electrophysiology catheter design was reported previously.^27^ This particular catheter employed frequencies ranging between 8 MHz and 15 MHz and boasted 4 cm to 6 cm of tissue penetration. Lateral resolution was reported as being 0.2 mm. The primary application of this integrated catheter was in tissue Doppler imaging and tissue synchronization imaging to determine the contractile wavefront propagation during electrophysiology testing. When it was used for monitoring lesion formation, although the formation of small bubbles was observed, no changes in the tissue characteristics were reported. IVUS utilizes either (1) a mechanical catheter that has a single transducer at the tip of a drive shaft that is advanced or withdrawn to scan the artery within an imaging sheath or (2) a synthetic aperture array catheter that has multiple, stationary transducers located around the catheter tip that are activated sequentially to produce cross-sectional images. These generate frequencies of between 20 MHz and 60 MHz and demonstrate a lateral resolution of around 0.2 mm.^28,29^ IVUS is primarily used in the diagnosis and monitoring of coronary artery disease.

OCT is a precise methodology able to reveal subendocardial structures. In this study, we have shown that OCT can visualize the AV node in fresh domestic porcine hearts. Clinical use of OCT may thus involve the visualization of AV nodal tissue during electrophysiology studies for guiding catheter ablation procedures and during the surgical repair of congenital heart disease. More specifically, OCT could be used to avoid AV node damage during slow pathway modifications, para-Hisian pathway ablations, or right anteroseptal pathway ablations. Additionally, AV node ablations in patients with chronic and persistent atrial fibrillation and/or flutter could be more precisely targeted and the time required for successful ablation might therefore be potentially shortened.

The implementation of this imaging modality in electrophysiology procedures will still necessitate the development of forward-imaging OCT probes and catheters that have OCT imaging capabilities in addition to standard ablation catheter properties. Work on the development of a hybrid catheter as well as the combining of an RFA catheter with a forward-viewing OCT system is currently being performed by different groups.^30,31^ This modality has the ability to monitor lesion formation in real time.

### Limitations

This study is a preliminary demonstration of the concept of using OCT to visualize the AV node. It was completed to show the detectability of the AV node using this method. Importantly, it includes a small sample size and lacks correlation between multiple observers due to its single-observation design. Furthermore, the manipulation of the tissues during imaging and marking with the pins was done by hand. Because of this, there is a small margin for offset, about 100 μm, to occur between the OCT images and the histology slides. However, considering the size of the AV node, the potential offset was not considered to be significant. Also, the time between OCT imaging and the preparation of the histology slides for each pig varied. This led to different levels of formalin exposure, dehydration, and retraction, which in the end resulted in some degree of difference in the appearance of the histology samples.

## Conclusions

We report the use of a novel imaging method that has the potential to guide ablation procedures around the AV node and improve procedural safety and efficiency. Further studies with multiple observers and including live animal experiments treated with catheter-based OCT systems will be needed to better assess the efficacy of this imaging technique and its potential for clinical use.

## References

[1] Saul PJ, Kanter RJ, Writing Committee (2016). PACES/HRS expert consensus statement on the use of catheter ablation in children and patients with congenital heart disease: Developed in partnership with the Pediatric and Congenital Electrophysiology Society (PACES) and the Heart Rhythm Society (HRS). Endorsed by the governing bodies of PACES, HRS, the American Academy of Pediatrics (AAP), the American Heart Association (AHA), and the Association for European Pediatric and Congenital Cardiology (AEPC).. Heart Rhythm..

[2] Kugler JD, Danford DA, Deal BJ (1994). Radiofrequency catheter ablation for tachyarrhythmias in children and adolescents.. N Engl J Med..

[3] An HS, Choi EY, Kwon BS (2013). Radiofrequency catheter ablation for supraventricular tachycardia: a comparison study of children aged 0-4 and 5-9 years.. Pacing Clin Electrophysiol..

[4] Ozaki N, Nakamura Y, Suzuki T (2018). Safety and efficacy of radiofrequency catheter ablation for tachyarrhythmia in children weighing less than 10kg.. Pediatr Cardiol..

[5] Jiang HE, Li XM, Li YH, Zhang Y, Liu HJ (2016). Efficacy and safety of radiofrequency catheter ablation of tachyarrhythmias in 123 children under 3 years of age.. Pacing Clin Electrophysiol..

[6] Kugler JD, Danford DA, Houston K (1997). Felix G.Radiofrequency catheter ablation for paroxysmal supraventricular tachycardia in children and adolescents without structural heart disease. Am J Cardiol..

[7] Scheinman MM, Huang S (2000). The 1998 NASPE prospective catheter ablation registry.. Pacing Clin Electrophysiol..

[8] Clague JR, Dagres N, Kottkamp H, Breithardt G, Borggrefe M (2001). Targeting the slow pathway for atrioventricular nodal reentrant tachycardia: initial results and long-term follow-up in 379 consecutive patients.. Eur Heart J..

[9] Spector P, Reynolds MR, Calkins H (2009). Meta-analysis of ablation of atrial flutter and supraventricular tachycardia.. Am J Cardiol..

[10] Collins KK, Dubin AM, Chiesa NA, Avasarala K, Van Hare GF (2006). Cryoablation versus radiofrequency ablation for treatment of pediatric atrioventricular nodal reentrant tachycardia: initial experience with 4-mm cryocatheter.. Heart Rhythm..

[11] Hanninen M, Yeung-Lai-Wah N, Massel D (2013). Cryoablation versus RF ablation for AVNRT: a meta-analysis and systematic review.. J Cardiovasc Electrophysiol..

[12] Thomas PE, Macicek SL (2016). Catheter ablation to treat supraventricular arrhythmia in children and adults with congenital heart disease: what we know and where we are going.. Ochsner J..

[13] Huang D, Swanson EA, Lin CP (1991). Optical coherence tomography.. Science..

[14] Bezerra HG, Costa MA, Guagliumi G, Rollins AM, Simon DI (2009). Intracoronary optical coherence tomography: a comprehensive review clinical and research applications.. JACC Cardiovasc Interv..

[15] Prati F, Jenkins MW, Di Giorgio A, Rollins AM (2011). Intracoronary optical coherence tomography, basic theory and image acquisition techniques.. Int J Cardiovasc Imaging..

[16] Fleming CP, Wang H, Quan KJ, Rollins AM (2010). Real-time monitoring of cardiac radio-frequency ablation lesion formation using an optical coherence tomography forward-imaging catheter.. J Biomed Opt..

[17] Fu X, Wang Z, Wang H, Wang YT, Jenkins MW, Rollins AM (2014). Fiber-optic catheter-based polarization-sensitive OCT for radio-frequency ablation monitoring.. Opt Lett..

[18] Fu X, Patel D, Zhu H (2015). Miniature forward-viewing common-path OCT probe for imaging the renal pelvis.. Biomed Opt Express..

[19] Jenkins M, Wade RS, Cheng Y, Rollins AM, Efimov IR (2005). Optical coherence tomography imaging of the Purkinje network.. J Cardiovasc Electrophysiol..

[20] Gupta M, Rollins AM, Izatt JA, Efimov IR (2002). Imaging of the atrioventricular node using optical coherence tomography.. J Cardiovasc Electrophysiol..

[21] Fleming CP, Ripplinger CM, Webb B, Efimov IR, Rollins AM (2008). Quantification of cardiac fiber orientation using optical coherence tomography.. J Biomed Opt..

[22] Ambrosi CM, Fedorov VV, Schuessler RB, Rollins AM, Efimov IR (2012). Quantification of fiber orientation in the canine atrial pacemaker complex using optical coherence tomography.. J Biomed Opt..

[23] Waller BD, Gering LE, Branyas NA, Slack JD (1993). Anatomy, histology and pathology of the cardiac conduction system: Part II.. Clin Cardiol..

[24] Tsimikas S, DeMaria AN (2012). The clinical emergence of optical coherence tomography: defining a role in intravascular imaging.. J Am Coll Cardiol..

[25] Vitulano N, Pazzano V, Pelargonio G, Narducci ML (2015). Technology update: intracardiac echocardiography—a review of the literature.. Med Devices (Auckl)..

[26] Hijazi ZM, Shivkumar K, Sahn DJ (2009). Intracardiac echocardiography during interventional and electrophysiological cardiac catheterization.. Circulation..

[27] Li XK, Pemberton J, Thomenius K (2007). Development of an electrophysiology (EP)-enabled intracardiac ultrasound catheter integrated with NavX 3-dimensional electrofield mapping for guiding cardiac EP interventions.. J Ultrasound Med..

[28] Mintz GS, Nissen SE, Anderson WD (2001). American College of Cardiology Clinical Expert Consensus Document on Standards for Acquisition, Measurement and Reporting of Intravascular Ultrasound Studies (IVUS). A report of the American College of Cardiology Task Force on Clinical Expert Consensus Documents. J Am Coll Cardiol..

[29] Mintz GS, Guagliumi G (2017). Intravascular imaging in coronary artery disease.. Lancet..

[30] Fleming CP, Wang H, Quan KJ, Rollins AM (2010). Real-time monitoring of cardiac radio-frequency ablation lesion formation using an optical coherence tomography forward-imaging catheter.. J Biomed Opt..

[31] Herranz D, Lloret J, Jimenez-Valero S, Rubio-Guivernau JL, Margallo-Balbas E (2015). Novel catheter enabling simultaneous radiofrequency ablation and optical coherence reflectometry.. Biomed Opt Express..

